# Enhancing clinical outcomes: Point of care ultrasound in the precision diagnosis and Management of Abdominal Aortic Aneurysms in emergency medicine: A systematic review and meta‐analysis

**DOI:** 10.1002/jcu.23850

**Published:** 2024-09-29

**Authors:** Eman E. Shaban, Yavuz Yigit, Baha Alkahlout, Ahmed Shaban, Amira Shaban, Benny Ponappan, Mohammed Abdurabu, Hany A. Zaki

**Affiliations:** ^1^ Department of Cardiology Al Jufairi Diagnosis and Treatment, MOH Qatar; ^2^ Department of Emergency Medicine Hamad Medical Corporation Doha Qatar; ^3^ Department of Internal Medicine Mansoura General Hospital Mansoura Egypt; ^4^ Department of Internal Medicine Mansoura University Hospital Mansoura Egypt; ^5^ Clinical Assistant Professor of Emergency Medicine College of Medicine, Qatar University (CMED ‐ QU) Doha Qatar

**Keywords:** abdominal aorta aneurysm, diagnostic accuracy, emergency department, point‐of‐care ultrasound, ruptured AAA detection

## Abstract

This meta‐analysis evaluates the efficacy of point‐of‐care ultrasound (POCUS) in diagnosing abdominal aortic aneurysm (AAA) in the emergency department (ED). A systematic search of PubMed, Cochrane Library, Scopus, and Google Scholar identified studies published until July 2024. Nine studies were included, revealing that POCUS is highly accurate in diagnosing AAA, with a pooled sensitivity of 98.33% and specificity of 99.84%. Additionally, data from three studies indicated that 24.5% of patients with positive AAA scans were diagnosed with ruptured AAAs. The results suggest that emergency physicians can accurately detect and manage AAA using POCUS, even with limited training.

## Graphical abstract

POCUS empowers rapid AAA detection and life‐saving action.

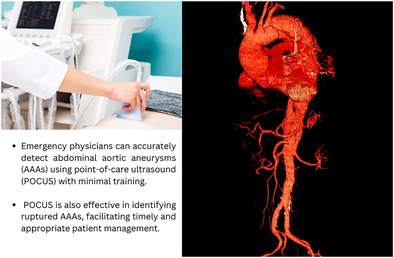

## 1 INTRODUCTION

An abdominal aortic aneurysm (AAA) is an abdominal aortic dilation of 3 cm or above.^1^ The incidence of this condition increases with age and is more common in men than women between the age of 74–84 years.^1^, ^2^, ^3^ If left untreated, it can progress to life‐threatening rupture that results in mortality rates of up to 90%.^4^ Moreover, 30‐day mortality rates of 36% and 39% have been reported after endovascular aneurysm repair and open repair of ruptured or asymptomatic AAA.^5^ As a result, emergency physicians (EPs) need to have access to an imaging modality that is readily available, noninvasive, accurate, and preferably portable to minimize mortality rates that occur if AAA is not diagnosed early.

The diagnosis of AAA is occasionally made by physical examination, which reveals a pulsatile mass left of the midline between the xyphoid process and the umbilicus. However, this diagnostic modality is moderately sensitive in detecting AAA, with one previous study suggesting that it has a sensitivity of 68% and a specificity of 75%.^6^ Furthermore, AAA diagnosis is often made incidentally by imaging modalities such as abdominal ultrasound and computed tomography. While CT scan remains the gold standard for diagnosing AAA, abdominal ultrasound is gaining interest in AAA detection due to its high sensitivity, which ranges between 95% and 100%, and specificity, which approaches 100%.^7^, ^8^ Generally, this diagnostic test is conducted in the radiology department. However, recent technological advancements have made ultrasound machines easier to operate, lighter, more mobile, and increasingly affordable. As a result, point‐of‐care ultrasound (POCUS), which provides real‐time information about unstable conditions such as AAA, has been adopted in emergency medicine.

One of the earliest reports on the use of POCUS in the emergency department (ED) reported that EPs were able to correctly diagnose the presence and size of AAAs in patients with pulsatile masses or unexplained abdominal pain.^9^ Since then, investigations into the role of POCUS in diagnosing AAA in the ED have been made. Therefore, this meta‐analysis was undertaken to examine the efficacy of POCUS in diagnosing and managing AAA in the ED.

## 2 METHODS

### 2.1 Literature search and information sources

This review article was carried out per the Preferred Reporting Items for Systematic Reviews and Meta‐analysis (PRISMA) guidelines.^10^ A systematic and comprehensive search for records published from inception until July 2024 was conducted in PubMed, Cochrane Library, Scopus, and Google Scholar databases. The search involved a combination of the following search terms: “Ultrasound,” “Point‐of‐care ultrasound,” “bedside ultrasound,” “Emergency department,” and “Emergency physicians.” Reference lists of articles retrieved from the mentioned electronic databases were scrutinized for further relevant records. In addition, we exempted duplicate records and gray literature since their inclusion would have reduced the statistical validity of our meta‐analysis. The detailed search strategy in each electronic database is outlined in Appendix A.

### 2.2 Eligibility criteria

Two independent reviewers screened full‐text records of all retrieved articles according to the following Patients, Intervention, Comparator, and Outcomes (PICO) criteria: P: Adult patients (≥18 years) suspected of having AAA, I: Bedside ultrasound or point‐of‐care ultrasound performed in the ED by EPs to diagnose AAA, C: Reference standards for diagnosing AAA such as computed tomography (CT), magnetic resonance imaging (MRI), formal ultrasound performed in the radiology department (RUS), exploratory laparotomy, or autopsy, O: Diagnostic accuracy and management outcomes. Moreover, only studies authored in English were included in this review.

Studies inconsistent with the aforementioned PICO criteria and those intended as conference proceedings, abstracts, editorials, case series, case reports, and narrative reviews were omitted. Furthermore, records where radiologists or certified vascular technicians performed POCUS in the ED were excluded. Studies with fewer than 10 participants were also eliminated since we suspected this low sample size would impair the statistical reliability of our meta‐analysis.

### 2.3 Data extraction and data items

Two impartial reviewers extracted data from the eligible studies using a standardized data extraction spreadsheet. The collected information included study ID (i.e., primary author, publication date, study location, and study design), specific details about the enrolled subjects (such as mean/median age, sex distribution, and sample size), ultrasound machine used, operators, reference standard(s), the definition of AAA, and the reported outcomes. Differences throughout this procedure were settled by consensus. However, an extra reviewer was engaged if a unanimous agreement was not obtained.

The endpoints of this meta‐analysis were categorized into diagnostic accuracy measures and management outcomes. Diagnostic accuracy measures pooled in our study were sensitivity and specificity. On the other hand, the role of POCUS in managing AAA was evaluated by pooling the number of ruptured AAAs observed in all positive scans of AAA.

### 2.4 Quality assessment

Since this research was intended to be a diagnostic review, the quality of each study was assessed using the Quality Assessment of Diagnostic Accuracy Studies (QUADAS) tool, which is available in the Review Manager program. The selection of this instrument was based on its validation in the assessment of diagnostic studies and its inclusion of several evaluation criteria (14 questions). The questions were categorized into bias assessment and applicability concerns. The bias assessment involved assessing patient selection, the index test, the reference standard, and the flow of timing. On the other hand, the criteria for applicability are comprised of patient selection, index test, and reference standard. Overall, a low risk of bias and concern was denoted by green, while a high and unclear risk of bias and concern was denoted by red and yellow, respectively.

### 2.5 Data synthesis

Data analysis was performed with two distinct statistical tools. STATA 16 software (Stata Corp., College Station, TX, USA) was used to carry out a precise meta‐analysis on the diagnostic accuracy of POCUS in detecting AAA. The DerSimonian–Laird random‐effects model was used to pool all the diagnostic measures (sensitivity and specificity) and account for the study variation. Interstudy heterogeneity was estimated using the *χ* ^2^ test, and the magnitude was assessed using the *I* ^2^ statistics. Heterogeneity was considered significant if the *χ* ^2^ *p*‐value was less than 0.05 and the *I* ^2^ value was greater than 50%.^11^, ^12^

The comprehensive meta‐analysis software (CMA V3) was used to pool data regarding the rates of AAA rupture in a one‐arm meta‐analysis. The pooled AAA rupture incidences detected on POCUS were presented in terms of the event rate (ER). All pooled results were presented with their corresponding 95% confidence intervals (CI). Furthermore, the meta‐analytic results were presented using forest plots. Publication bias in the diagnostic measures was investigated via a visual inspection of funnel plots. Symmetrical plots indicated the absence of publication bias, while asymmetrical plots denoted the presence of bias.

## 3 RESULTS

### 3.1 Study selection

**FIGURE 1:**
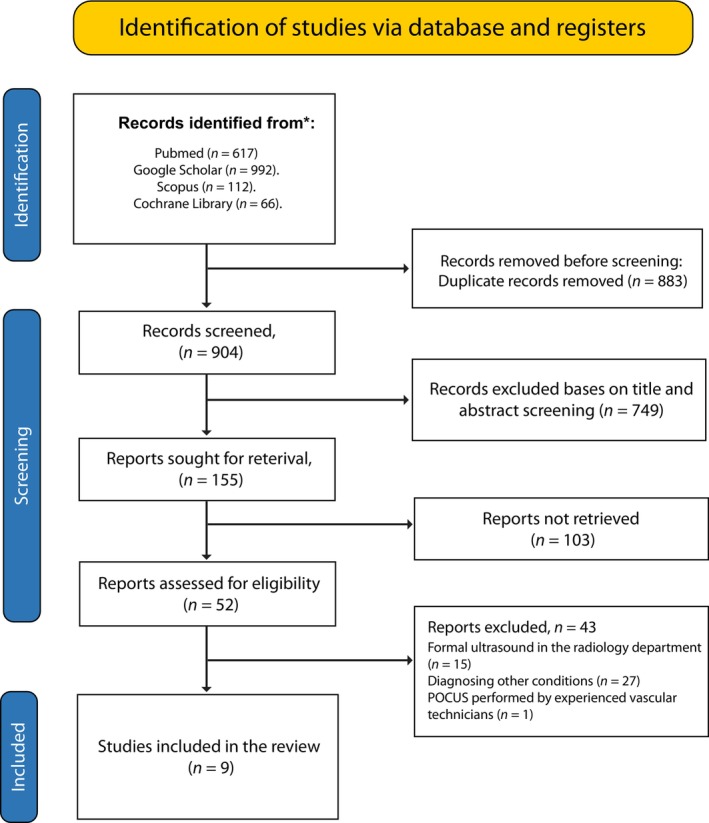
PRISMA flow diagram for study selection.

Our electronic database search returned a total of 1787 potential records. Unfortunately, the manual scrutiny of bibliographies did not identify any additional studies. A thorough review of these studies led to the omission of 883 duplicate records. The remaining records had their titles and abstracts thoroughly reviewed, of which 749 were omitted as they were considered irrelevant to our study objective. Of the remaining 155 studies, we did not retrieve 103 since they were designed as case reports, case series, editorials, narrative reviews, conference abstracts, or meta‐analyses. The remaining 52 records were retrieved for in‐depth scrutiny using the predefined PICO criteria. After the scrutiny, only nine were eligible for inclusion. The other studies were excluded as follows: 15 enrolled participants who underwent formal ultrasound in the radiology department, 27 reported the use of POCUS in diagnosing other conditions associated with abdominal pain, and 1 reported the use of ultrasound in the ED but performed by experienced vascular technicians (Figure 1).

### 3.2 Summary of study characteristics

A summary of the nine included studies^13^, ^14^, ^15^, ^16^, ^17^, ^18^, ^19^, ^20^, ^21^ is shown in Table 1. Four studies reported the mean age of enrolled participants, which ranged from 66 to 73 years. The other five did not report the mean age of enrolled participants. The level of ultrasound training and the POCUS machines used to detect AAA varied across the studies. Furthermore, there was variation in the reference standards for AAA diagnosis. Seven studies reported the use of multiple modalities as the reference standards. These modalities included CT, RUS, MRI, angiography, laparotomy, or autopsy. Eraybar and colleagues reported a single imaging modality (Contrast‐enhanced thoracic and abdominopelvic CT) as the reference standard.^13^ On the other hand, Lanoix et al.^20^ used a radiologist's interpretation of POCUS findings as their reference modality. All the studies reported the sensitivity and specificity of POCUS in diagnosing AAA in the ED. However, only three studies reported the role of POCUS in detecting ruptured AAA.

**TABLE 1:** Study Characteristics.

Study ID	Study Design	Study location	Patient characteristics			Ultrasound machine and views	Definition of AAA	Operators	Reference tests	Outcomes		
			Sample (n)	M/F	Mean age (years)					SEN (%)	SPEC (%)	Diagnosis of AAA rupture, n (%)
Eraybar et al.^13^	Prospective study	Turkey	133	78/47	67	Siemens Digital Color Doppler Ultrasound SIUI Apogee 3500 device	Aortic diameter ≥3 cm	EPs that had finished a proper training course on US usage.	Contrast‐enhanced thoracic and abdominopelvic CT.	100	91	5 (4)
Dent et al.^14^	Prospective study	United Kingdom	119	NR	73	Toshiba Nemio	Aortic diameter >3 cm	EPs training in emergency US and supervised by a more experienced physician.	CT, formal US, laparotomy, or post‐mortem	96.3	100	12 (46)
Tayal et al.^15^	Prospective study	United States	125	68/57	66	Shimadzu 400 and Shimadzu 450 gray‐scale ultrasound machines	Aortic diameter >3 cm	Senior EM residents and EM attending physicians with a minimum introductory education on US and had performed at least 50 emergency US prior to the study.	RUS, abdominal CT, abdominal MRI, or laparotomy.	100	98	NR
Constantino et al.^16^	NR	United States	238	NR	NR	Sonosite 180plus or Siemens Adara	Aortic diameter >3 cm	3rd year EM residents who learned emergency ultrasound during their residency and had performed at least 150 emergency US scans.	CT, MRI, and angiography in the radiology department.	94	100	5 (14.7)
Spampinato et al.^17^	Retrospective study	Italy	844	418/426	71	ESAOTE MyLab XPRO30	NR	EPs who participated in a basic course on POCUS, 10% with advanced POCUS certification, and a POCUS instructor.	RUS or abdominal CT	80	100	NR
Jones et al.^18^	Descriptive analysis study	New Zealand	58	NR	NR	B‐K Medical Cheetah 2003 Ultrasound system	NR	3 EPs and one critical care fellow	RUS, CT, laparotomy, or autopsy.	98	100	NR
Kuhn et al.^19^	NR	Australia	68	NR	NR	Aloka Flexus model SSD‐1100 scanner	Aortic diameter >3 cm	EPs and EM residents with 3 or more years of postgraduate experience who attended a 3‐day US training course.	RUS, CT, angiography, or laparotomy	100	100	NR
Lanoix et al.^20^	Prospective study	United States	NR	NR	NR	1986 Diasonics DRF400 ultrasound machine	Aortic diameter >3 cm	EPs who underwent a 4‐hour US training program	Review of US findings by a radiologist with fellowship training in US.	100	100	NR
Rowland et al.^21^	Prospective study	Australia	221	NR	NR	Aloka SSD 1100	NR	EPs who underwent a 3‐day training course on US.	Laparotomy, Abdominal CT scan, RUS, or Autopsy	100	100	NR

Abbreviations: CT, computed tomography; EPs, emergency physicians; MRI, magnetic resonance imaging; NR, not reported; RUS, formal ultrasound performed in the radiology department; US, ultrasound.

### 3.3 Diagnostic accuracy of POCUS

**FIGURE 2:**
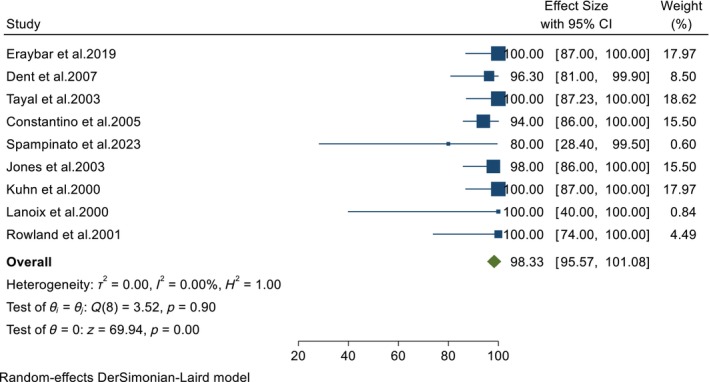
Forest plot showing the pooled sensitivity of POCUS in diagnosing AAA in the ED.

**FIGURE 3:**
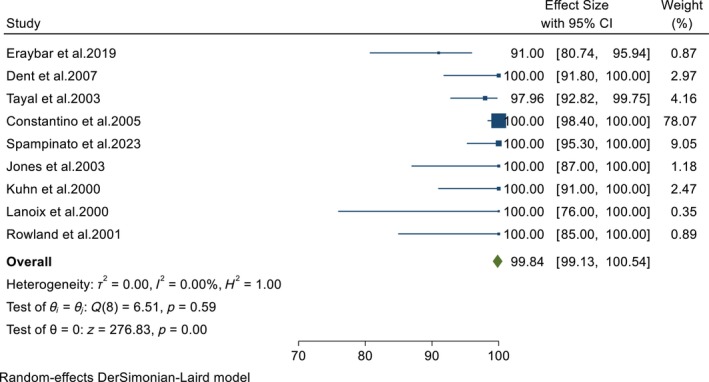
Forest plot showing the pooled specificity of POCUS in diagnosing AAA in the ED.

All studies in this meta‐analysis examined the accuracy of POCUS in diagnosing AAA in the ED. The pooled results from these studies showed that POCUS was highly sensitive and specific in diagnosing AAA in the ED (98.33%, 95% CI: 95.57–100 and 99.84%, 95% CI: 99.13–100, respectively) (Figures 2 and 3). Furthermore, the test for heterogeneity did not show any variation in the pooled diagnostic measures (*I* ^2^ = 0%; *p* = 0.90 for sensitivity and *I* ^2^ = 0%; *p* = 0.59 for specificity), meaning that the diagnostic accuracy of POCUS for AAA was consistent across the included studies.

### 3.4 Ruptured AAA

**FIGURE 4:**
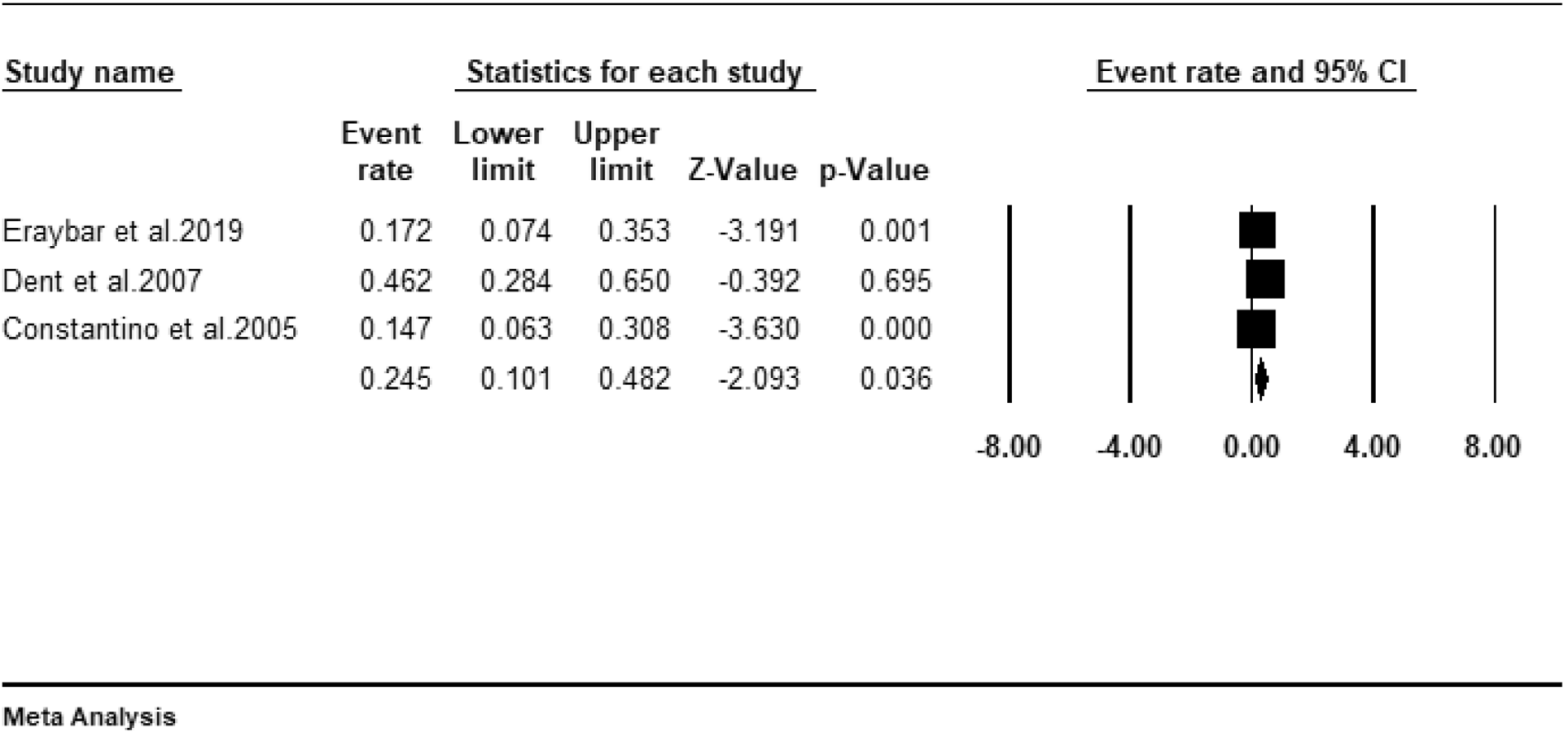
Forest plot showing the pooled incidence of ruptured AAA in patients identified to have AAA using POCUS.

AAA rupture, a highly fatal aortic pathology, was reported in three studies involving 490 patients suspected of AAA. The data from these studies showed that POCUS detected AAA in 89 patients, of which our pooled results demonstrated that ruptured AAAs represented 24.5% (95% CI: 10.1–48.2) of the positive scans (Figure 4).

### 3.5 Methodological quality

**FIGURE 5:**
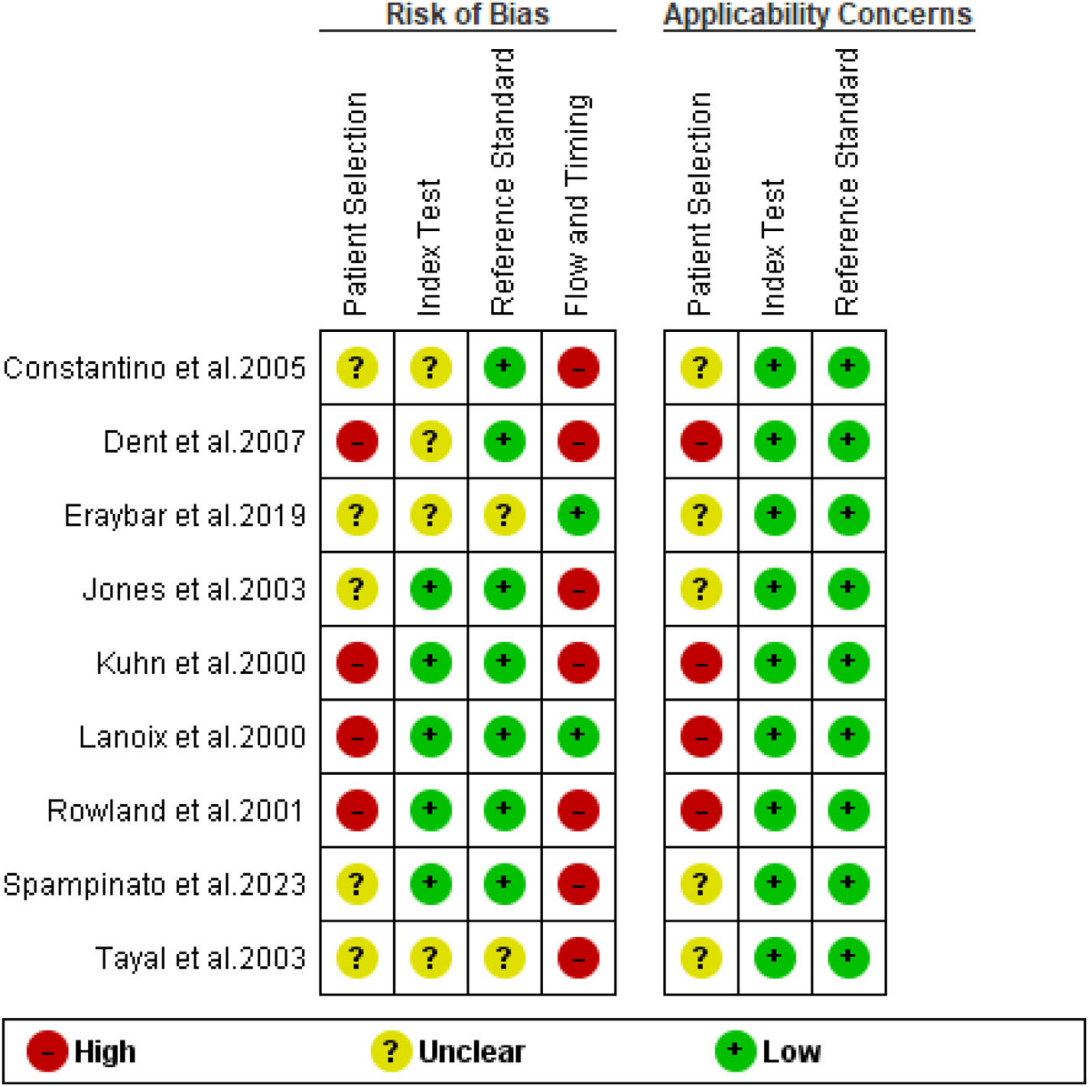
Risk of bias and applicability concerns summary.

Figure 5 represents a summary of the methodological evaluation using QUADAS‐2. The evaluation showed that four of the included studies had a higher risk of selection bias. The other five studies had unclear risk of selection bias since they did not provide any information regarding sampling method. In addition, seven of the included studies demonstrated a high risk of bias concerning flow and timing. This bias was associated with the fact the studies employed more than one reference standard, which was considered a limitation.

### 3.6 Publication bias

**FIGURE 6:**
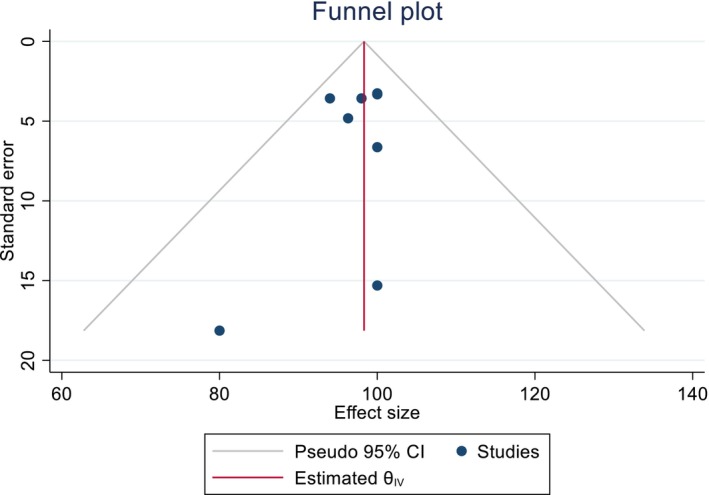
Funnel plot showing the absence of publication bias in the pooled sensitivity of POCUS in detecting AAA.

**FIGURE 7:**
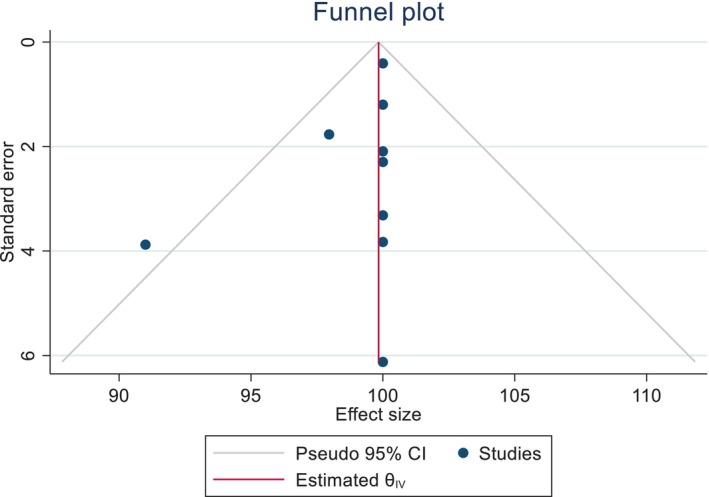
Funnel plot showing the absence of publication bias in the pooled specificity of POCUS in detecting AAA.

Using funnel plots, we were able to investigate the publication bias in the diagnostic accuracy of POCUS. After a visual inspection of the plots, we did not find any publication in the sensitivity and specificity of POCUS in diagnosing AAA (Figures 6 and 7).

## 4 DISCUSSION

Over the years, several modalities have been used to diagnose AAA. At one time, cross‐table lateral radiographs were the most commonly used diagnostic tests for AAA, but they were found to be insufficient in detecting this highly catastrophic disease.^22^ Abdominal CT, which is highly sensitive and specific, has also served as an alternative modality for detecting AAA.^23^, ^24^ However, this modality is expensive, time‐consuming, requires highly trained operators, and needs the transfer of unstable patients from the area of resuscitation. MRI, on the other hand, does not offer any advantages over CT in detecting AAA and has more logistical disadvantages, particularly in unstable patients.^25^ In addition, arteriography, which is capable of providing information on the vascular anatomy, has been used in AAA detection, but since it is cumbersome, it has been relegated to selected stable patients.^26^ Recently, technological advancements in ultrasound machines have made ultrasound easier to perform and interpret the findings at the patient's bedside.^27^ As a result, ultrasound has been incorporated into emergency medicine to provide real‐time information about several pathologies at the patients' point of care. Therefore, this review has investigated the role of POCUS in diagnosing and managing AAA among patients presenting to the ED.

Our pooled analysis has shown that POCUS performed in the ED achieves an acceptable sensitivity and specificity in detecting AAA. This finding corresponds with the results of previous meta‐analyses. For instance, a meta‐analysis of 11 studies reported that ultrasound performed by non‐radiologists (EPs, primary care physicians, or surgical residents) had a pooled sensitivity of 98% and specificity of 99% for AAA diagnosis.^28^ Similarly, Rubano and colleagues found bedside ultrasound was highly accurate in diagnosing AAA in the ED (pooled sensitivity and specificity of 99% and 98%, respectively).^29^ Considering these findings, it is safe to say that EPs can accurately use ultrasound to diagnose AAA at the patients' bedside. Furthermore, POCUS also offers several benefits beyond high diagnostic accuracy. First, bedside tests take a few minutes to perform, meaning that they can reduce ED overcrowding.^30^ Second, since patients do not need to be transferred to the radiology department, POCUS can reduce the imaging workload of radiologists.^31^ Third, the performance of POCUS by EPs rather than certified sonographers can ensure the 24‐h availability of this vital service. Finally, POCUS ultrasound can address the requirements of patients presenting with the classical triad of signs and symptoms.

Interestingly, the study characteristics show that most POCUS examinations were performed by inexperienced EPs with limited ultrasound training. Therefore, our pooled results suggest that EPs with limited training and experience in POCUS training can accurately diagnose AAA. However, it is uncertain how much training in POCUS is required to accurately diagnose AAA. A previous study by Hoffman and colleagues suggested that certified ED sonographers with experience of less than 3 years were significantly less likely to detect AAA than those with higher experience.^32^ In contrast, one of the studies included in our review suggested that EPs who underwent a 3‐day ultrasound training course could detect the presence or absence of AAA with high sensitivity and specificity.^21^ Similarly, Lanoix and colleagues found that a 4‐hour ultrasound training was sufficient to rule in and out AAA in the ED.^20^ However, these studies included a convenience sample, meaning that the investigators might have enrolled patients who were easy to scan, thus introducing selection bias. Therefore, further investigation in prospective studies with consecutive patients is needed to ascertain the minimum POCUS training required to make an accurate diagnosis of AAA in the ED.

Our meta‐analysis also suggests that POCUS might have a role in identifying patients with ruptured AAA. A recent retrospective study showed that prehospital use of POCUS in detecting ruptured AAA is associated with a reduction in time to treatment, increased operability, and improved 30‐day survival rates.^33^ Therefore, timely diagnosis of ruptured AAA using POCUS in the ED might impact patient care. For instance, Dent and colleagues found that out of the 14 patients identified to have ruptured AAAs, surgical repair was performed in four, and the other 10 were deemed inappropriate for surgical or endovascular repair.^14^ On the other hand, Shuman et al.^34^ investigated the diagnostic value of ultrasound performed by radiologists called to the ED before the arrival of patients suspected of AAA and found that ultrasound could correctly identify 21 of 22 ruptured AAAs requiring immediate surgical repair. Therefore, detecting ruptured AAA using ultrasound might help identify patients suitable for repair and those who may be spared futile attempts at resuscitation.

While POCUS might be accurate in detecting AAA, it has several limitations that must be addressed. First, excess bowel gas and obesity make it challenging to visualize the aorta using ultrasound. Previous research has shown an association between increased waist circumference and variability in ultrasound measurements of the abdominal aorta.^35^ Therefore, a low‐frequency curvilinear transducer of 2–5 MHz is recommended to visualize the aorta. Moreover, a further decrease in frequency may occasionally be needed to improve penetration.^36^ On the other hand, limited visualization of the abdominal aorta due to bowel gas might be overcome by placing patients in the lateral decubitus position while providing gentle pressure on the abdomen to displace the bowel on the side.^36^, ^37^ Second, ultrasound is not entirely accurate, meaning misdiagnoses are likely to occur. These misdiagnoses might be associated with several factors. Since the aorta and inferior vena cava (IVC) travel parallel,^38^ sonographers are likely to misidentify the abdominal aorta for IVC if they sweep too far to the patient's right in the parasagittal plane. Moreover, paraaortic nodes are situated around the aorta. Most large nodes are situated anteriorly. However, some nodes are located posteriorly and might displace the aorta anteriorly away from the vertebral body.^39^ These nodes, if not examined carefully, may be mistaken for the aorta due to their location and appearance. Finally, the various techniques for measuring the aorta diameter might limit the accuracy of ultrasound in detecting AAA.^39^ However, further studies are warranted to support this claim.

### 4.1 Limitations

It is important to take into account the inherent limitations of the present meta‐analysis when evaluating its conclusions. First, the literature search was restricted to items written in English. Therefore, data from non‐English records that would have been utilized to enhance our scientific research and increase the statistical value of this meta‐analysis was purposely removed; therefore, our study was prone to selection bias. Second, some of the included studies recruited patients using convenience sampling, implying that they were at a greater risk of selection bias, which impacted their diagnostic accuracy. Consequently, this selection bias was extended to our analyses. Third, the included studies had small sample sizes (fewer than 1000 subjects); hence, our statistical analysis was prone to small sample size bias. Finally, retrospective studies were also included in the meta‐analysis, implying that the strength of data acquired from this research is reduced owing to biases surrounding this study design.

## 5 CONCLUSION

In summary, this meta‐analysis has shown that EPs can accurately undertake POCUS to detect AAA even with limited training. Moreover, EPs can utilize POCUS to identify patients with ruptured AAA, which allows appropriate management to be undertaken. Unfortunately, POCUS examinations performed by EPs are still subject to misdiagnosis and are limited in patients with bowel gas and obesity. Therefore, until these issues are resolved, POCUS cannot replace CT as the gold standard but can be considered an adjunct imaging modality.

## Data Availability

The data that support the findings of this study are available from the corresponding author upon reasonable request.

## APPENDIX A SEARCH STRATEGY

### A.1 PUBMED

**[All fields]** (ultrasound OR ultrasonography OR abdominal ultrasound OR bedside ultrasound OR Point of care ultrasound OR POCUS) AND (Abdominal aortic aneurysm OR aortic aneurysm OR abdominal aneurysm) AND (Emergency room OR emergency medicine OR emergency department OR ED OR ER OR emergency physicians OR emergency residents).

**[Titles/Abstract]** (ultrasound OR ultrasonography OR abdominal ultrasound OR bedside ultrasound OR Point of care ultrasound OR POCUS) AND (Abdominal aortic aneurysm OR aortic aneurysm OR abdominal aneurysm) AND (Emergency room OR emergency medicine OR emergency department OR ED OR ER OR emergency physicians OR emergency residents).

### A.2 GOOGLE SCHOLAR

**[with all of the words]** (Bedside ultrasound OR Point of care ultrasound OR POCUS) AND (Abdominal aortic aneurysm) AND (emergency department OR ED).

**[with at least one of the words]** (ultrasound OR ultrasonography OR abdominal ultrasound OR bedside ultrasound OR Point of care ultrasound OR POCUS) AND (Abdominal aortic aneurysm OR aortic aneurysm OR abdominal aneurysm) AND (Emergency room OR emergency medicine OR emergency department OR ED OR ER OR emergency physicians OR emergency residents).

### A.3 SCOPUS

(ultrasound OR ultrasonography OR abdominal ultrasound OR bedside ultrasound OR Point of care ultrasound OR POCUS) AND (Abdominal aortic aneurysm OR aortic aneurysm OR abdominal aneurysm) AND (Emergency room OR emergency medicine OR emergency department OR ED OR ER OR emergency physicians OR emergency residents).

### A.4 COCHRANE LIBRARY

(ultrasound OR ultrasonography OR abdominal ultrasound OR bedside ultrasound OR Point of care ultrasound OR POCUS) AND (Abdominal aortic aneurysm OR aortic aneurysm OR abdominal aneurysm) AND (Emergency room OR emergency medicine OR emergency department OR ED OR ER OR emergency physicians OR emergency residents).
